# Identification of survival-associated alternative splicing events and signatures in adrenocortical carcinoma based on TCGA SpliceSeq data

**DOI:** 10.18632/aging.102924

**Published:** 2020-03-26

**Authors:** Ning Xu, Zhi-Bin Ke, Xiao-Dan Lin, Fei Lin, Shao-Hao Chen, Yu-Peng Wu, Ye-Hui Chen, Yong Wei, Qing-Shui Zheng

**Affiliations:** 1 Departments of Urology, The First Affiliated Hospital of Fujian Medical University, Fuzhou 350005, China

**Keywords:** alternative splicing, adrenocortical carcinoma, TCGA, survival

## Abstract

Objective: To explore the correlations among alternative splicing (AS), splicing factors (SF) and survival outcome in adrenocortical carcinoma (ACC) patients.

Results: A total of 92 ACC patients were included. Univariate analysis identified 3919 AS events significantly associated with overall survival. Lasso method followed by multivariate analysis revealed that the prognostic capacity of these survival-related AS events is satisfactory. Interestingly, we found that the area under the curve (AUC) of AA, AD, AP and RI were more than 0.9, indicating that these four types of AS were of great significance. Independent prognostic analysis showed that only the risk score was the independent risk factor of ACC survival. Finally, we constructed an interesting interaction network between AS and SF.

Conclusions: This is the first and most comprehensive study to explore the aberrant AS variants in ACC, which might provide novel insights into molecular mechanism of ACC.

Methods: The transcriptome data, clinical information and Percent Spliced In (PSI) values of the ACC were obtained from TCGA database and TCGA SpliceSeq data portal. Lasso method and uni/multivariate Cox regression analysis were used to identify survival-related AS events and develop multi-AS-based signatures. The relationship between AS events and SFs was also investigated.

## INTRODUCTION

As a rare but greatly aggressive malignancy, the prognosis of adrenocortical carcinoma (ACC) is rather poor [1, 2]. Surgical excision currently remains the preferred treatment for ACC in patients with localized lesion [3]; however, the five-years overall survival is only 15–44% even if the tumor is completely removed [4]. Although there have been numerous studies exploring ACC, the specific molecular mechanism has not been fully elucidated.

As the most important post-transcriptional regulatory process, alternative splicing (AS) modifies more than 90% of human genes [5, 6], contributing greatly to protein diversity and complexity [7]. Numerous physiological and pathological behaviors in the human body are related to AS, including angiogenesis, proliferation, hypoxia, etc [5, 8, 9]. It was reported that survival predictors of gene expression were consistently inferior to AS-based survival predictors [10]. There were emerging data revealing that abnormal AS events were involved in carcinogenesis, immunologic escape, cancer progression and metastasis [6, 10–12]. However, little work has been devoted to investigate the role of AS in ACC.

In this study, a total of 92 patients with ACC from the Cancer Genome Atlas (TCGA) database were enrolled to comprehensively profile genome-wide AS events, which play a vital role in the development of ACC. Further, uni/multivariate Cox regression analysis and Lasso regression analysis were used to identify survival-related AS events and develop multi-AS-based signatures. We also explored the relationship between AS events and clinical feature using clinical data from TCGA database. Moreover, AS related splicing factors (SF) were identified and functional enrichment analyses were performed.

## RESULTS

### Clinical characteristics and integrated AS events

**Figure 1:**
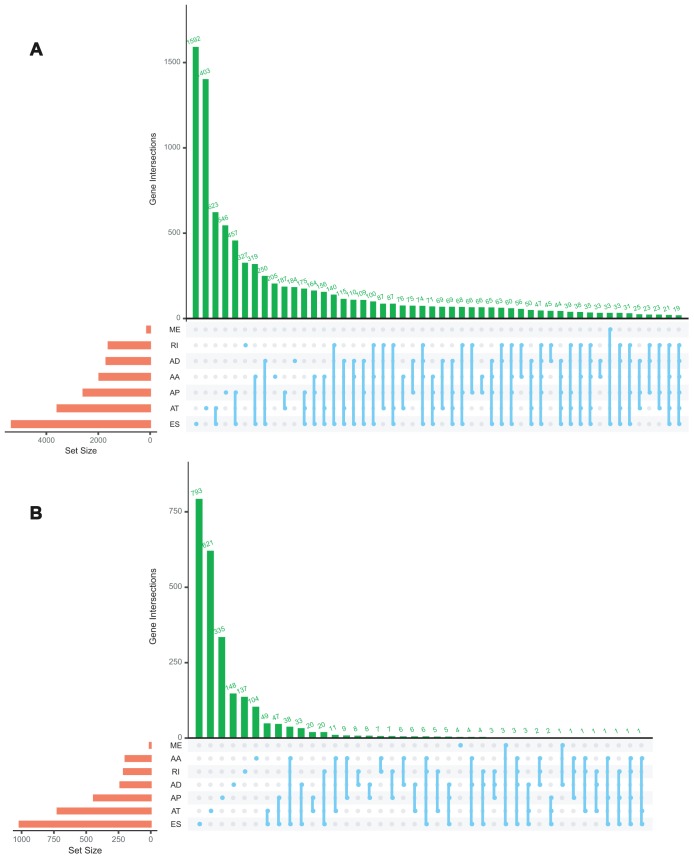
Upset plot of interactions among seven types of all AS events (**A**) and survival-associated AS events (**B**) in ACC.

A total of 92 ACC patients from TCGA database were included in the present study. The clinicopathological characteristics of these 92 cases were summarized in Table 1. The AS events were comprehensively analyzed. Intersections among these seven types of AS events and quantitative analysis of these interactive sets were presented by the UpSet plot (Figure 1A). These data suggested that a single gene could possess several types of mRNA splicing events and ES was the predominant type in ACC cohort whereas ME was rare.

**Table 1:** Clinicopathological characteristic of 92 patients with adrenocortical carcinoma from TCGA database.

**Clinicopathological characteristics**	**Value**
Gender, n(%)	
Female	60(65.2)
Male	32(34.8)
TCGA stage, n(%)	
Stage I	9(9.7)
Stage II	44(47.8)
Stage III	19(20.7)
Stage IV	18(19.6)
Unknown	2(2.2)
T stage, n(%)	
T1	9(9.7)
T2	49(53.3)
T3	11(12)
T4	21(22.8)
Unknown	2(2.2)
N stage, n(%)	
N0	80(87.0)
N1	10(10.8)
Unknown	2(2.2)
Survival, n(%)	
Yes	59(64.1)
No	33(35.9)

### Survival associated AS events

**Figure 2:**
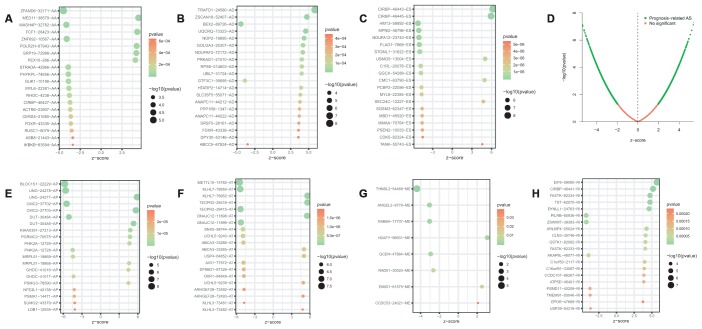
Bubble plots of top 20 significant prognostic AS events in AA (**A**), AD (**B**), ES (**C**), AP (**E**), AT (**F**), ME (**G**) and RI (**H**) type, respectively. Volcano plots of prognostic AS events (**D**). AA, alternate acceptor site; AD, alternate donor site; AP, alternate promoter; AT, alternate terminator; ES, exon skip; ME, mutually exclusive exons; RI, retained intron.

Univariate Cox regression analysis between AS events and OS was conducted to determine the survival associated-AS events. A total of 3919 AS events were significantly associated with OS (P<0.05). Consequently, one gene might contain two or more AS events that were remarkably related to the OS, and ES was the most common survival associated event. The UpSet plot was generated to visualize the intersecting sets of genes and splicing types vividly (Figure 1B). The top 20 (if available) significant survival associated AS events of each type were selected and visualized in Figure 2. The distributions of AS events significantly associated with OS were displayed in Figure 2D.

### Construction and evaluation of the prognostic AS signature

**Figure 3:**
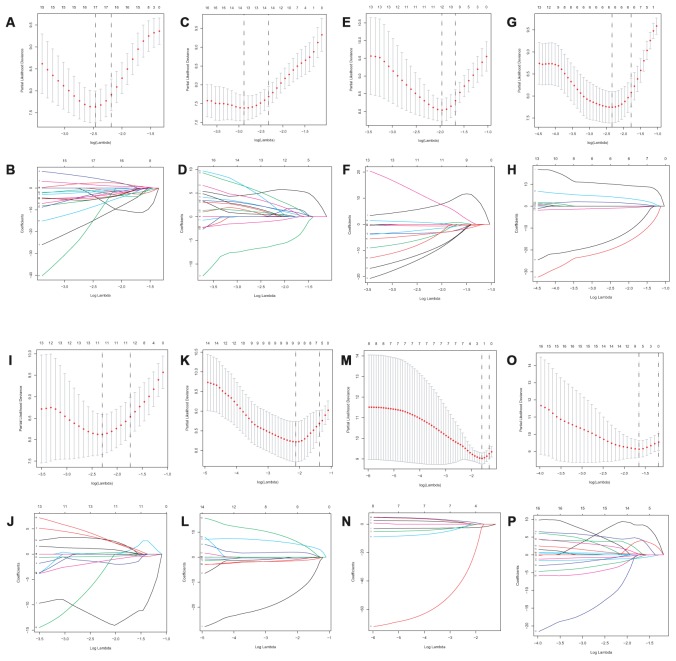
**Lasso regression analysis of survival-associated AS events.** AA cohort (**A**, **B**), AD cohort (**C**, **D**), ES cohort (**E**, **F**), the whole cohort (**G**, **H**), AP cohort (**I**, **J)**, AT cohort (**K**, **L**), ME cohort (**M**, **N**) and RI cohort (**O**, **P**).

**Figure 4:**
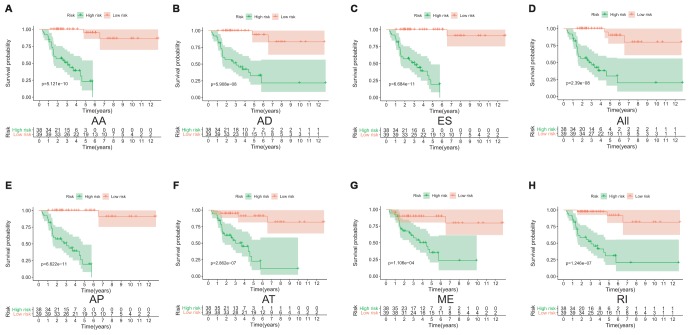
**The Kaplan-Meier curves analysis of overall survival between the low-risk patients and high-risk patients.** AA cohort (**A**), AD cohort (**B**), ES cohort (**C**), the whole cohort (**D**), AP cohort (**E**), AT cohort (**F**), ME cohort (**G**) and RI cohort (**H**).

**Figure 5:**
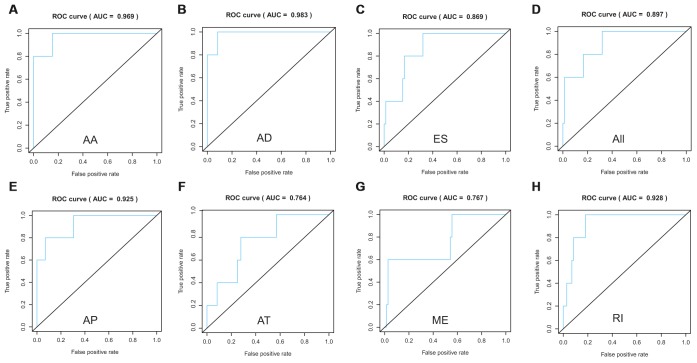
**ROC curves to evaluate the predictive power of each prognostic signature.** AA cohort (**A**), AD cohort (**B**), ES cohort (**C**), the whole cohort (**D**), AP cohort (**E**), AT cohort (**F**), ME cohort (**G**) and RI cohort (**H**).

**Figure 6:**
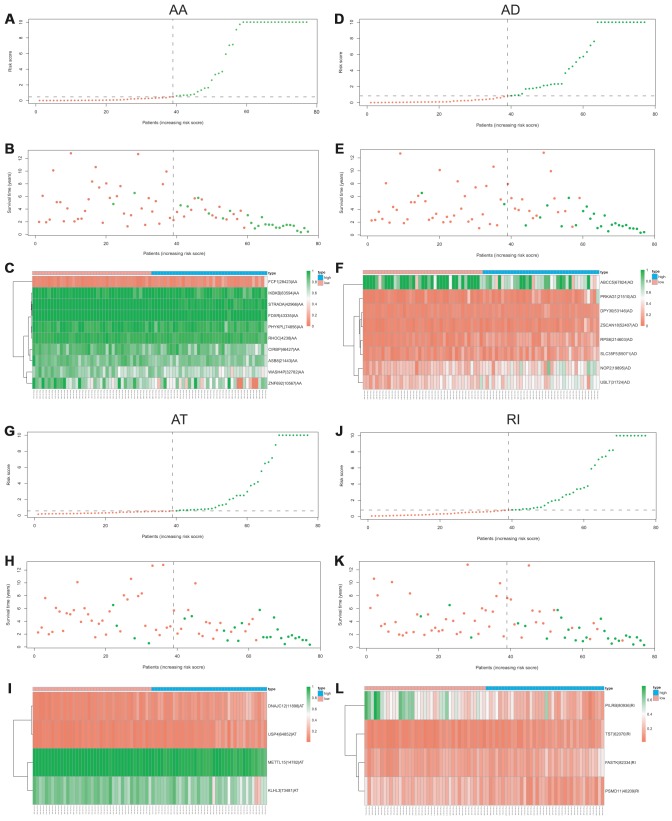
**The distribution of risk score, the distribution of survival time, the expression Heatmap of the most four significant prognostic signatures with AUC > 0.9.** AA cohort (**A**–**C**), AD cohort (**D**–**F**), AT cohort (**G**–**I**), and RI cohort (**J**–**L**).

Lasso method followed by multivariate Cox regression analyses were performed to evaluate the prognostic capacity of these survival related AS events. The results of Lasso regression analysis including seven types of AS events and all AS events were presented in Figure 3. Using Lasso regression analysis, we selected the most highly correlated AS events. Next, the particular AS events were selected and risk scores were calculated based on multivariate Cox regression model. The detailed information of particular AS events in the eight prognostic models was shown in Table 2. Moreover, ACC patients were stratified into low-risk and high-risk groups based on the median value of the risk score as cut off. The Kaplan-Meier curves were employed to demonstrate the survival probability variation of patients in the low-risk and high-risk groups. The results showed that survival time differed notably between these two groups in each type and the whole cohort (Figure 4). The ROC curves were generated to evaluate the predictive power of each prognostic signature. Finally, four prognostic signatures with AUC ≥ 0.9 were selected for subsequent analysis. The results revealed that risk score of AD performed the greatest prognostic power with an AUC of 0.983, followed by risk score of AA with an AUC of 0.969 and risk score of RI with an AUC of 0.928 (Figure 5). The detailed information of corresponding splicing pattern of the candidate AS events, living status as well as survival time ranked by the distribution of risk score was displayed (Figure 6).

**Table 2:** Multivariate Cox analysis of prognostic alternative splicing predicting overall survival.

	**gene symbol**	**Spliceseq ID**	**AS type**	**coef**	**HR**	**HR.95L**	**HR.95H**	**pvalue**
AA	WASH4P	32782	AA	-14.32072025	6.03E-07	4.69E-10	0.000776507	8.85E-05
	FCF1	28423	AA	10.52242085	37138.92339	26.67193939	51713510.98	0.004385308
	ZNF692	10567	AA	-5.156531463	0.00576165	0.000481514	0.0689422	4.66E-05
	STRADA	42966	AA	-49.80983757	2.33E-22	4.63E-36	1.17E-08	0.001972983
	PHYKPL	74856	AA	9.080654761	8783.715377	0.268598401	287245402.7	0.086875639
	RHOC	4238	AA	-23.48714955	6.30E-11	9.57E-21	0.415281483	0.041735412
	CIRBP	46427	AA	-11.51432883	9.99E-06	1.55E-08	0.006424444	0.000483316
	FDXR	43335	AA	-89.66982198	1.14E-39	1.11E-57	1.17E-21	2.26E-05
	ASB8	21443	AA	-8.054295393	0.000317734	9.34E-07	0.10803302	0.006764598
	IKBKB	83594	AA	-12.55412881	3.53E-06	6.50E-11	0.191660267	0.024010337
AD	NOP2	19895	AD	5.78087704	324.0432647	21.79721757	4817.313818	2.69E-05
	PRKAG1	21510	AD	7.942308557	2813.848948	35.46032682	223284.6285	0.000372286
	RPS6	214603	AD	10.79652418	48850.70931	58.21054288	40995869.17	0.001671678
	UBL7	31724	AD	5.487061825	241.5464576	4.520725761	12906.04523	0.006867221
	SLC35F5	55071	AD	9.405234339	12151.8213	54.51807766	2708583.414	0.000650922
	DPY30	53146	AD	13.83177834	1016400.818	0.119714777	8.62943E+12	0.089281102
	ABCC5	67824	AD	-2.297745789	0.100485103	0.014785187	0.682930561	0.018773482
	ZSCAN18	52407	AD	12.12172017	183821.448	25.95655419	1301803178	0.007364346
AP	DUT	30484	AP	-5.620022604	0.003624559	7.82E-05	0.168012062	0.004088329
	PGRMC2	70575	AP	6.70571319	817.060538	46.33192005	14408.81194	4.66E-06
	PSMG3	78590	AP	11.30833143	81497.81036	662.6229713	10023638.45	4.11E-06
	PSMA1	14471	AP	-24.41434895	2.49E-11	5.66E-19	0.00109977	0.006556697
AT	METTL15	14782	AT	-29.33321587	1.82E-13	1.85E-20	1.79E-06	0.000356241
	DNAJC12	11898	AT	9.26480082	10559.7071	30.50774066	3655053.161	0.001898066
	USP4	64852	AT	16.05609602	9398832.355	334.0948681	2.6441E+11	0.002127869
	KLHL3	73481	AT	-2.259390566	0.104414099	0.005981425	1.822693335	0.121496101
ES	STOML1	31622	ES	-9.160094929	0.000105153	4.91E-07	0.022507891	0.000820894
	USMG5	13004	ES	35.97110089	4.18842E+15	2920100.949	6.01E+24	0.000826184
	C1RL	20076	ES	-28.40353921	4.62E-13	6.34E-19	3.37E-07	3.72E-05
	GGCX	54288	ES	-9.137141275	0.000107594	5.07E-07	0.022819339	0.000828759
	MMAA	70764	ES	-21.66172696	3.91E-10	5.37E-14	2.85E-06	1.81E-06
	PSEN2	10033	ES	-23.24041531	8.07E-11	2.43E-20	0.267974754	0.037737792
ME	THNSL2	54469	ME					
RI	FASTK	82334	RI	15.24965535	4196054.973	1668.459188	10552776755	0.000134968
	TST	62070	RI	10.79258808	48658.80626	0.097705043	24232929547	0.106858488
	PILRB	80936	RI	-5.625477112	0.003604843	0.000114349	0.113642411	0.001397614
	PSMD11	40209	RI	-5.425612937	0.004402367	2.48E-06	7.81580743	0.155222621
All	CIRBP	46443	ES	13.99695993	1198953.832	0.067535731	2.12849E+13	0.100277799
	BLOC1S1	22229	AP	-25.72792057	6.71E-12	3.20E-20	0.001404383	0.008491906
	TRAFD1	24580	AD	6.050752744	424.4323987	1.54910273	116288.5183	0.034618673
	METTL15	14782	AT	-23.56412231	5.84E-11	6.33E-19	0.005385426	0.011794019
	HM13	58892	ES	-1.957454311	0.14121746	0.011251275	1.772454358	0.129386117

### Stratification survival analysis with the prognostic signatures

**Figure 7:**
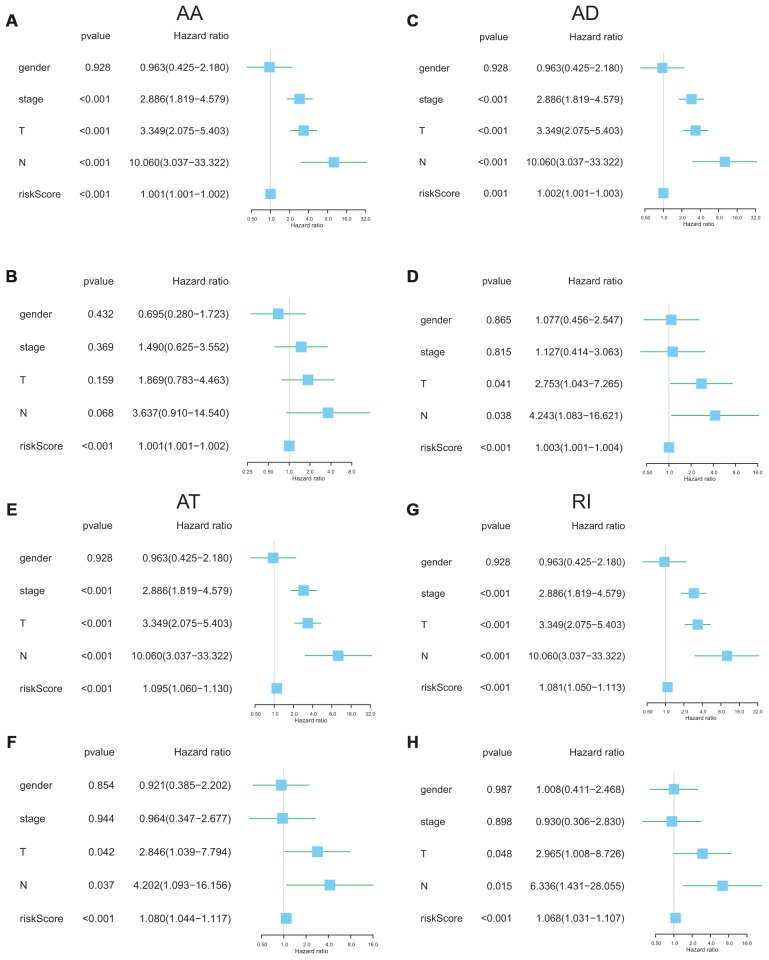
**Independent prognostic analysis determining predictors of overall survival.** AA cohort (**A**, **B**), AD cohort (**C**, **D**), AT cohort (**E**, **F)**, and RI cohort (**G**, **H**).

The following clinical parameters including gender, T stage, N stage and clinical stage were integrated into the uni/multivariable Cox regression analysis. Univariate Cox regression analysis indicated that several clinical features could predict poorer survival of ACC patients including high T stage, high N stage and high clinical stage and high risk-score. However, multivariate Cox regression showed that the risk score was the only independent risk factor of ACC survival (Figure 7). These results revealed that the risk scores with an AUC ≥ 0.9 were independent prognostic indicators after adjusted by the clinicopathological characteristics.

### Functional enrichment analysis of AS events related SF genes

**Figure 8:**
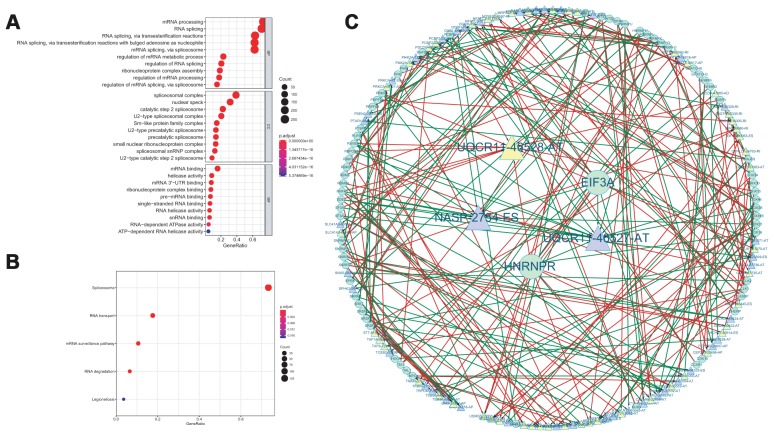
GO functional enrichment analysis (**A**) and KEGG pathway analysis (**B**) of AS events related SF genes. The interaction network between SF and AS events (**C**).

Functional enrichment analysis was performed for the sake of revealing the potential mechanisms of corresponding genes (known as SFs) with survival-associated AS events. The biological process terms of these genes were mainly related to “mRNA processing”, “RNA splicing”, “RNA splicing via transesterification reactions”. “RNA splicing via transesterification reactions with bulged adenosine as nucleophile”. “Spliceosomal complex”, “nuclear speck”, “catalytic step 2 spliceosome” were the three most significant cellular component terms. For molecular function, “mRNA binding”, “helicase activity” and “mRNA 3’-UTR binding” were three most enriched categories (Figure 8A). KEGG analysis revealed a total of 5 remarkably enriched pathways, including “spliceosome”, “RNA transport”, “mRNA surveillance pathway”, “RNA degradation”, and “Legionellosis” (Figure 8B).

### Construction of potential splicing regulatory network

Then, a correlation network between the expression of SFs and the PSI values of survival-associated AS events was conducted. As shown in Figure 8C, there were a total of 42 down-regulated AS events (purple triangles), 50 up-regulated AS events (yellow triangles) and 67 SFs in this network. In this network, we selected the five most significant nodes as the hub AS events or hub SFs according to the degree, including one upregulated AS event (UQCR11-46528-AT), two downregulated AS events (UQCR11-46527-AT and NASP-2754-ES) and two SF (EIF3A and HNRNPR).

## DISCUSSION

It has been reported that there was an extremely high incidence of splicing defects in carcinogenesis and that RNA splicing modulators might be a promising target for cancer treatment [13–15]. Alternative splicing allows cells to generate diverse [13, 14] mRNA by modifying mRNA isoforms [16]. There have been numerous studies exploring the important role of AS in various malignant tumors, including colorectal cancer [17], renal cell carcinoma [18], breast cancer [19] and so on. Gray et al. [20]. found a novel splicing event which showed both an allelic expression imbalance and preferential splicing for one of the alleles in ACC samples. Previous studies demonstrated that the activation of spliced X-box protein 1(XBP1) - mRNA splicing might be one of the important molecular mechanisms of mitotane in the treatment of ACC [21–23]. Besides, Kroiss et al. [22]. found that combination of bortezomib and carfilzomib with low-dose mitotane could be used to treat ACC effectively by increasing XBP1-mRNA splicing. Freddi et al. [24]. explored the expression of splice variants of growth hormone-releasing hormone receptor (GHRH-R) in human primary adrenocortical tumors and found that splice variants might be a key pathogenic factor in adrenal carcinogenesis. However, limited studies focused on the AS events in occurrence and progression of ACC.

As far as we know, this is the first study to date lucubrating the aberrant alternative spliced variants in patients with ACC using high throughput data. In this study, we systematically explored the prognostic value of AS events in ACC patients for the first time. A total of 3919 AS events, which were significantly associated with survival outcome, were identified by univariate Cox regression analysis. Next, Lasso regression and multivariate Cox regression analysis were preformed to constructed eight the predictive significances, including seven types of AS and all AS, respectively. These ACC patients from TCGA database were then divided into low- and high-risk groups according to the risk score. Kaplan-Meier analysis demonstrated that the difference of overall survival between the low-risk patients and high-risk patients were significant. Besides, the results showed that AUC of AA, AD, AP and RI were more than 0.9, indicating that these four types of AS were of great significance in predicting survival outcome in ACC patients. As a consequence, these four types of AS were screened to further analysis. However, as is known to us, there were few studies related to AA, AD, AD and RI in ACC. Future study into these four AS types in ACC might have noticeable research value. Given the high prevalence of splicing defects in tumor, these prognostic AS events identified in this study could be novel therapeutic targets in ACC treatment.

As is known to us, splicing factors were one of the vital regulated factors of AS events [25]. Changes of expression or sequence mutations of SFs may affect splicing events [26]. To elucidate the underlying mechanism of AS events in the survival of ACC patients, we performed GO and KEGG enrichment analysis for the SFs related significantly to AS events. According to the results, “mRNA processing”, “RNA splicing” and “RNA splicing via transesterification reactions” were the three most significant biological process. “Spliceosomal complex”, “nuclear speck”, “catalytic step 2 spliceosome” were the three most significant cellular component terms. For molecular function, “mRNA binding”, “helicase activity” and “mRNA 3’-UTR binding” were three most enriched categories. The GO results revealed that these SFs have a significant relationship with splicing. KEGG analysis demonstrated that spliceosome is the most significant pathway among these SFs. Besides, we also established an interaction network between AS events and SF. In this network, we selected the five most significant nodes as the hub AS events or hub SF according to the degree, including one upregulated AS event (UQCR11-46528-AT), two downregulated AS events (UQCR11-46527-AT and NASP-2754-ES) and two SF (EIF3A and HNRNPR). These new hub SF genes and AS events were not reported previously, which need further validation.

There were several limitations in this study. Firstly, we merely analyzed the survival-associated AS events of ACC using TCGA database due to no additional external cohort concerning splicing data. Secondly, this is a retrospective study with limited number of patients. Further study with larger sample size is warranted.

## CONCLUSIONS

In summary, this is the first and most comprehensive study to explore the aberrant alternative spliced variants in patients with ACC. These identified survival-related alternative spliced events and the interesting interaction network between AS and SF might provide novel insights into molecular mechanism of ACC. Further researches are needed to investigate the role of AS in ACC.

## MATERIALS AND METHODS

### Data collection and preprocessing

The transcriptome data and clinical information of the ACC cohort were downloaded from The Cancer Genome Atlas (TCGA) database (https://portal.gdc.cancer.gov/). Percent Spliced In (PSI) values for AS events of 33 various tumor types and available adjacent normal samples, have been loaded into TCGA SpliceSeq data portal (https://bioinformatics.mdanderson.org/TCGASpliceSeq/) [13]. The AS-related data in TCGA SpliceSeq is of high quality [13]. The data, download procedure and operating environment in TCGA SpliceSeq have been widely used to explore survival-associated AS events and signatures of various malignant tumors [12, 13]. We obtained AS events with PSI value of ACC from the TCGA SpliceSeq data portal. There were seven types of AS events, including retained intron (RI), exon skip (ES), mutually exclusive exons (ME), alternate promoter (AP), alternate donor site (AD), alternate terminator (AT) and alternate acceptor site (AA).

### Identification of survival-related AS events

Univariate Cox regression analysis was carried out to evaluate the association between AS events and overall survival (OS). UpSet plot and volcano Plot was used to present the survival-related AS events based on the seven types of AS events. Besides, the bubble chart was used to summarize the top 20 all AS events, top 20 RI events, top 20 ES events, top 8 ME events, top 20 AP events, top 20 AD events, top 20 AT events and top 20 AA events.

### Prognostic risk scores calculation and survival analysis

Lasso regression analysis was further conducted to screen highly correlated AS events and avoid overfit the models constructed subsequently. Multivariate Cox regression analysis was applied to calculate the prognostic risk scores for OS prediction based on seven types of AS events. All patients were divided into high-risk group and low-risk group according to the median risk score. Kaplan–Meier curves were used to demonstrate the survival probability variation between low-risk and high-risk subgroups. Receiver operating characteristic (ROC) curve was constructed and the area under the curve (AUC) was also calculated. Further, prognostic evaluation of the risk score was performed using risk curve. The expression heatmap, distribution of risk score and survival time of related signature were then visualized.

### Independence of prognostic signature

The following clinicopathological characteristic from TCGA database including gender, T stage, N stage and clinical stage were subjected to subsequent analysis. Univariate and multivariate Cox regression analysis was performed to evaluate whether the final AS prognostic signature with an AUC ≥ 0.9 was an independent risk factor of OS.

### Functional enrichment analyses

The list of splicing factor (SF) genes was collected from the SpliceAid 2 database (http://193.206.120.249/splicing_tissue.html) [14] and was shown in Supplementary Table 1. The expression profiles of SF genes were downloaded from TCGA database. To further illustrated the underlying mechanisms of AS in ACC, we identified corresponding SF genes of AS events. Functional enrichment analyses, including Gene Ontology (GO) analysis and the Kyoto Encyclopedia of Genes and Genomes (KEGG) pathway analysis, for these SF genes was performed. The results of GO analysis were presented by three parts including biological processes (BP), molecular functions (MF), and cellular component (CC). Both GO analysis and KEGG analysis was conducted using R x64 3.6.1 software.

### Construction of splicing regulatory network

Spearman test was conducted to analyze the correlation between the expression of SF genes and PSI values of survival-associated AS events. P value < 0.001 and correlation coefficient > 0.6 was considered statistically significant. Besides, we constructed an interaction network of AS and SF. The plug-in Molecular Complex Detection (MCODE) was used to select most significant modules from the interaction network. The most significant interaction network was then visualized by Cytoscape software (version 3.4.0). Furthermore, the top five hub nodes were selected according to the connection degree from this interaction network.

## Supplementary Material

Supplementary Table 1
